# Exploring the strategies used by managers to support midwives experiencing adverse events in a selected district of Gauteng province

**DOI:** 10.4102/hsag.v31i0.3268

**Published:** 2026-05-18

**Authors:** Rebotile V. Morobe, Thifhelimbilu I. Ramavhoya, Mamare A. Bopape

**Affiliations:** 1 Department of Nursing, Faculty of Health Science, University of Limpopo, Polokwane, South Africa

**Keywords:** adverse event, district, midwife, obstetric units, strategies

## Abstract

**Background:**

Globally, adverse events (AEs) remain a major patient safety concern, as they are unintended injuries resulting from healthcare management rather than from the patient’s condition. Managerial support is essential to help midwives cope. Limited research exists on strategies that managers use to support midwives following AEs.

**Aim:**

To explore and describe strategies used by managers to support midwives who experience AEs in obstetric units of selected district in Gauteng province.

**Setting:**

Three public hospitals in Gauteng province, South Africa.

**Methods:**

Qualitative, exploratory, descriptive and contextual design was used. Twelve managers were purposively selected through non-probability sampling. Data were gathered using semi-structured interviews, lasting 30 min – 45 min. A pilot study conducted tested interview process and analysed using Tesch’s coding.

**Results:**

Findings revealed three themes and six sub-themes. Support included assistance with incident reporting; assessment of AEs; creation of supportive work environment; provision training; emotional support; and follow-up sessions.

**Conclusion:**

Managerial support is critical in mitigating emotional and professional impact on midwives. A 24-hour counselling service was recommended.

**Contribution:**

This study provide evidence on managers’ support strengthening emotional and professional systems. The findings will improve policies, leadership practices and training of midwives.

## Introduction

In South Africa, the Department of Health (DoH) mandates the delivery of quality healthcare services and the reduction of adverse events (AEs), as outlined in the *Nursing Act No. 33 of 2005* and the *National Health Act No. 61 of 2003* (Mulaudzi et al. 2021). The study examined the strategies used by managers to support midwives during AEs. By overcoming these barriers, incident reporting not only identifies and rectifies safety concerns but also catalyses a positive safety culture in midwifery (Wain, Clarke & Wall 2024).

Adverse events in maternal health care remain a significant concern worldwide, especially in low- and middle-income countries, including those in sub-Saharan Africa (Gombau-Giménez et al. 2022). Midwives, as primary caregivers in maternal and reproductive health, often find themselves at the forefront of these events, experiencing emotional, psychological and professional repercussions. International studies have shown that midwives working in high-pressure environments frequently experience burnout, stress and moral distress, factors that not only affect their well-being but also contribute to AEs in patient care (Hamed & Konstantinidis 2022; Kim et al. 2021, Kumah 2025; Isaac et al. 2024). Despite global and regional efforts to improve maternal health outcomes, the emotional support given to midwives following AEs remains inadequate in many settings (Sidhu et al. 2020).

Midwives play a vital role in achieving this mission by providing safe, competent and ethical care. However, despite their training and adherence to professional guidelines, AEs such as maternal deaths, birth injuries or neonatal complications still occur, leading to emotional trauma, professional scrutiny and even litigation (Cankaya, Erkal Aksoy & Dereli Yılmaz 2021). This issue is particularly pronounced in public hospitals across Gauteng province, a densely populated and economically active region that draws patients from across South Africa and neighbouring countries, thereby straining an already overburdened healthcare system and causing inequality of care (Nchabeleng 2022; Ngene, Khaliq & Moodley 2023). Midwives in Gauteng’s public hospitals frequently provide care under strenuous conditions, with high patient loads, limited resources and increasing community expectations. In such settings, the emotional and psychological aftermath of AEs is profound. Xu et al. (2025) suggested that inadequate support for midwives following AEs leads to unresolved grief, job dissatisfaction, low morale, burnout and poor retention rates. While midwives may find temporary relief in peer support, the absence of structured managerial strategies exacerbates their distress and contributes to further errors and patient safety risks.

Research has shown that effective managerial support can mitigate the negative effects of AEs by fostering a culture of learning rather than blame (Luo 2025). Scoping reviews and international studies have highlighted the importance of institutional strategies, including debriefing sessions, psychological support, structured incident reporting systems and mentoring (Evans et al. 2023; Liukka et al. 2020; Lou et al. 2022). A qualitative study conducted by Thapa et al. (2021) in Sweden reported similar findings: a lack of managerial support for midwives jeopardises care in the obstetric unit. However, in many sub-Saharan countries, including South Africa, such systems are underdeveloped or poorly implemented, and managers are often unavailable because of administrative duties, leaving midwives feeling abandoned during their most vulnerable moments (Morobe, Ramavhoya & Bopape 2024).

The introduction of standardised protocols, such as the Patient Safety Incident (PSI) reporting system by the Gauteng DoH, aims to enhance accountability and learning from AEs. However, practical implementation remains inconsistent. Many healthcare workers still perceive AE reporting as punitive, particularly when reporting mechanisms lack transparency, and managerial support is minimal. In addition, the psychological effects of interacting with unfamiliar or poorly introduced technologies have been reported to increase the risk of AEs, further emphasising the need for supportive managerial strategies in the wake of such events. Given the increasing frequency and complexity of AEs in maternal health care, the emotional toll on midwives and the current gaps in support systems within healthcare institutions, this study aims to explore the strategies used by managers to support midwives who experience AEs in selected public hospitals in Gauteng province. Understanding strategies such as open lines of communication, a supportive environment, identifying AEs and preventing their recurrence is crucial not only for the well-being of midwives but also for improving maternal healthcare outcomes and maintaining quality care within the system.

## Research methods and design

### Research design

A qualitative exploratory descriptive design was chosen as appropriate for the study to explore the strategies managers use to support midwives experiencing AEs in obstetric units within a selected district of Gauteng province.

### Study setting

This study was conducted in a selected district of Gauteng province, South Africa. Gauteng, formerly known as Pretoria-Witwatersrand-Vereeniging, comprises three metropolitan municipalities: Ekurhuleni, Johannesburg and Tshwane. As the country’s economic hub, Gauteng province has a population of over 15 million. The selected district includes academic hospitals and serves a diverse population of approximately 12.27m. The study was conducted in obstetric units at hospitals in this district. A media statement by Puchert (2025) reported that AEs rose from 4170 in 2019 to 7386 in 2024 in Gauteng province. These selected hospitals had a higher incidence of AEs than academic hospitals, averaging 40 births per day and reporting a total of 252 AEs between 2020 and 2021.

### Population and sampling

The target population of this study comprised managers employed at district hospitals in Tshwane. A total of three district hospitals were selected. These hospitals were selected because they had higher AEs rates than the surrounding academic hospitals, thereby facilitating the identification of participants who met the inclusion criteria. A non-probability, purposive sampling method was used to select the 12 managers.

### Inclusion and exclusion criteria

The inclusion criteria for selecting the 12 managers were:

- Employed in the Tshwane district with over 2 years of experience as a manager.
- Registered with the South African Nursing Council (SANC) as a midwife.
- Placed in the obstetric unit, willing to participate.

Managers were selected because the researcher assumed they had addressed AEs in their managerial roles. Those who did not meet the criteria were excluded.

### Accessing participants

Permission to conduct the study was secured through formal applications to the district offices and the research committees of the selected facilities. Participants were recruited using the above-mentioned inclusion criteria; their names were recorded under pseudonyms, in accordance with the *Protection of Personal Information Act* (POPIA). The list of participants was kept under lock and key to prevent third-party access. The researcher scheduled appointments at each manager’s convenience, explained the study’s purpose and objectives, and ensured that the participants voluntarily signed consent forms.

### Pilot study

The researcher conducted a pilot study, a small-scale preparatory study, to test research protocols and data collection tools, to analyse and refine them before the main study began (Saunders 2024). Two managers from a non-participating district hospital who met the selection criteria were purposively selected as AEs are common in public facilities, and managers would have similar experience with AEs. The researcher revised some questions to improve clarity and align them with the study objective. The pilot study’s findings were not included in the main study.

### Data collection

Data were collected at the three district hospitals in Tshwane. An initial meeting with hospital managers at each facility was held as an information session to explain the study’s purpose. At these meetings, the researcher confirmed that the research would not disrupt hospital operations. The researcher was introduced to the research committees and unit managers. The researcher scheduled appointments with participants and explained the purpose of the study. All participants who met the eligibility criteria and agreed to participate in the study received information letters (Flanagan & Beck 2024). A follow-up appointment for data collection was scheduled based on each participant’s availability. Before gathering data, participants signed informed consent forms after being informed of their right to withdraw from the study at any time without consequences.

A semi-structured interview guide was developed to facilitate data collection. Interviews were conducted in English, the medium of communication, in a private room to ensure privacy and minimise disruption. The researcher conducted one-to-one interviews using a voice recorder and documented field notes on nonverbal cues. Participants were alerted when the voice recorder was switched on and off. One central open-ended question was asked of each participant: ‘Which strategies are you using as managers to support midwives in case there is an adverse event in the unit?’ This was followed by probing questions based on participants’ responses, to ensure in-depth data were obtained. Another probing question was ‘Was it important to support midwives, after an adverse event, and why?’ Interviews with participants lasted 30 min – 40 min, depending on the depth of the responses. During the interviews, the researcher used bracketing, intuitive and reflective remarks, and set aside preconceived ideas and beliefs about the phenomenon. During the intuitive phase, the researcher followed the interview guide’s questions. Lastly, reflection was conducted when participants offered interesting responses, and the researcher encouraged them to elaborate by asking questions such as ‘Tell me more, or did you mean that?’

The recordings were transcribed within 24 h, while the researcher still had a rich memory of the interviews. The transcribed data were stored in a password-protected document accessible only to the researcher to protect participants’ confidentiality and anonymity. Data collection continued until data saturation was reached; no further information emerged from the 10th participant. However, two further interviews were conducted to confirm saturation. The entire data collection process was conducted from 05 March 2023 to 27 May 2023.

### Data analysis

Interview audio recordings were transcribed verbatim and reviewed repeatedly to ensure all relevant information was captured. Field notes and transcripts were analysed by the researcher after each collection day. Following the analysis, the data and transcripts were provided to the independent coder. A consensus meeting was held with the independent coder to agree on the identified themes and sub-themes. Direct quotations from participants are presented in italics. Tesch’s inductive, descriptive open coding techniques (Creswell & Plano-Clark 2024) were followed, as outlined next.

#### Step 1. Reading through all the collected data

The researcher repeatedly read through verbatim transcripts to gain a general sense of the overall content. Initial ideas, including the meaning, were jotted down. This was done to identify data segments and ideas to understand their meanings.

#### Step 2. Reduction of collected data

The researcher reduced the collected data to codes and examined the frequency of concepts in the transcripts. The researcher listed all emerging topics, grouping them by similarity; those that did not belong to any group were grouped separately.

#### Step 3. Asking about the meaning of the collected data

Researchers analysed interview transcripts, drawing on recurring themes. The questions focused on the most interesting concepts: ‘What is this about and what is the underlying message?’ These questions helped the researcher understand the meaning, which in turn informed coding.

#### Step 4. Abbreviation of data to codes

Thoughts that arose from this question were jotted down on the side of the book. Once the researcher had completed the task for each respondent, similar topics were compiled into columns, arranged by major and unique topics. Data that did not match any topic were identified as ‘residues’.

#### Step 5. Development of themes and sub-themes

Themes and sub-themes were developed from the coded data and all grouped texts related to one another or sharing the same meaning.

#### Step 6. Comparison of codes, topics and themes for duplication

The researcher had to start from scratch to check for duplication and determine whether any codes, themes or topics needed to be revised.

#### Step 7. Grouping of themes and sub-themes

Columns were drawn in which preliminary groupings of themes and sub-themes were made, prior to the meeting with the independent coder. A consensus meeting was held to identify themes and sub-themes derived from the data.

#### Step 8. Recoding

There was no need to recode.

### Ensuring trustworthiness

To ensure the study’s trustworthiness, the researcher rigorously applied the criteria of credibility, confirmability, transferability and dependability as outlined by Polit and Beck (2019), Creswell and Plano-Clark (2023), and Brink, Van der Walt and Van Rensburg (2022).

Credibility refers to confidence in the truthfulness of the findings of the study. This was enhanced by engaging participants over time, with interviews lasting 30–40 min each. In addition, an independent coder was involved in the data analysis to further enhance credibility. Confirmability was ensured by maintaining an audit trail, which allowed the researcher to demonstrate objectivity and minimise bias by employing bracketing techniques throughout the research process (Houser & Oja 2025). Transferability refers to the extent to which the findings are applicable to similar contexts. This was addressed by providing a comprehensive description of the managers’ experiences and the study’s context (Creswell & Plano-Clark 2023). Dependability was ensured by repeating the study procedures, asking the same questions of the same participants in a similar context, and obtaining consistent findings (Houser & Oja 2025; Polit & Beck 2018). The involvement of an independent coder during data analysis enhanced the study’s reliability.

### Ethical considerations

Ethical clearance to conduct this study was obtained from the University of Limpopo (No. TREC/588/2022:PG) and the Tshwane Research Committee (GP_202301_040). Access to the facilities was granted for data collection. Informed consent was obtained from respondents. Privacy and anonymity (using pseudonyms) were maintained throughout the study. Participants were informed of their right to withdraw from the study at any time if they felt uncomfortable, without prejudice. Consent forms and questionnaires were prepared in English, and participants voluntarily signed them to take part in the study.

## Results

The results of the strategies managers used to support midwives revealed three themes and six sub-themes. Data were collected from 12 managers at the three selected facilities.

### Biographical data of participants

Twelve managers took part in the study, as shown in Table 1. Two were aged between 36 and 42 years, four were between 43 and 49 years, and the remaining six were over 50 years of age. The educational levels of the 12 participants were as follows: two held basic qualifications in midwifery, five had post-basic qualifications in advanced midwifery, and five held various degrees in nursing. All 12 participants had more than 10 years of nursing experience, indicating extensive knowledge and expertise in managing maternal health conditions. This indicates that maternal health care was managed by experts in maternity services.

**TABLE 1:** Demographic profile of participants.

Respondent	Age (in years)	Gender	Experience	Qualification
1	39	Female	14 years	Advanced Midwifery
2	62	Female	38 years	Bachelor of Nursing and Hons
3	45	Female	23 years	Bachelor of Nursing
4	55	Female	22 years	Masters in Nursing
5	47	Female	23 years	Advanced Midwifery
6	56	Female	23 years	Advanced Midwifery
7	39	Female	15 years	Advanced Midwifery
8	46	Female	24 years	BCUR and Hons
9	48	Female	26 years	Masters
10	40	Female	16 years	Advanced Midwifery

*Source*: Morobe, R.V., 2024, ‘Development of a model to support midwives experiencing adverse events in obstetric units of the Tshwane District, Gauteng Province’, Doctoral thesis, University of Limpopo.

Table 2 presents the results, organised into three themes and six sub-themes. The main themes identified by managers in the results are as follows: clinical, psychological and organisational support.

**TABLE 2:** Summary of themes and sub-themes on strategies used by managers to support midwives.

Main theme	Sub-themes
1. Clinical support strategies	1.1 The completion of incident report forms 1.2 Supportive incident assessment as a management strategy
2. Psychosocial support strategies	2.1 Reassurance as a form of emotional support 2.2 Provision of follow-up
3. Organisational and capacity-building support strategies	3.1 Training of midwives 3.2 Creating a conducive environment

*Source:* Morobe, R.V., 2024, ‘Development of a model to support midwives experiencing adverse events in obstetric units of the Tshwane District, Gauteng Province’, Doctoral thesis, University of Limpopo.

### Theme 1: Clinical support strategies

Clinical support strategies provided by managers to midwives following AEs include completing incident report forms (PSI), conducting a supportive assessment of the incident and providing post-AE follow-up. Participants reported that when an AE occurs, managers assess the nature, extent and severity of the event, including its potential medico-legal implications. Managers also indicated that they support midwives throughout the documentation process and ensure appropriate follow-up, as shown in Table 2.

#### Sub-theme 1.1: The completion of incident report forms

Managers support midwives following AEs by guiding and overseeing the completion of the PSI. An effective incident reporting system underpins safe clinical practice within healthcare organisations by facilitating the reporting, documentation (Hamdan et al. 2023) and analysis of unplanned incidents that result in AEs. Participants described managerial guidance on the steps required to complete the PSI form after an AE. One participant emphasised that accurate, comprehensive documentation of what occurred, the contributing factors and the timing of the event ensures factual reporting (Falcone et al. 2022) and supports informed decision-making about subsequent actions. One participant mentioned:

‘An incident form is completed by the midwife, guided by the manager, and reports what happened chronologically, including the times when the incident occurred. As managers, we must oversee the process and protect the midwives and the hospital from legal issues that may arise in public hospitals. The report must be objective, avoiding opinions or assumptions, and must outline the immediate actions taken. The report must be signed and submitted to management for review. Midwives must know that completing an incident form (PSI) prevents further occurrences of AEs.’ (Manager 2, Female, 62 years)

Another participant concurred and added that the DoH has developed an incident reporting form to standardise AE reporting:

‘The department has developed what is called the Patient Safety Incident (PSI) form, which makes it easy for us to assist midwives in completing it as required by the National Department. In doing so, we don’t have to go around in circles because the correct information will be completed, and the midwife will know exactly what is required.’ (Manager 11, Female, 49 years old)

Another participant reported that the standardised PSI form is intended to help the affected party capture the required concise information about the incident. However, participants reported that midwives were reluctant to document incidents, viewing this as a form of punishment by management. A participant confirmed, saying:

‘I would like to begin by noting that the National Department of Health has established a standardised form so that we can all follow the same when reporting AEs. Midwives drag their feet when they have to write an incident. You have to follow up on them for a long time, as they take PSI as a sign of incompetence. Their peers talk about these in the corridors, and even fail to understand that it also protects you and is not a punishment but a learning curve.’ (Manager 3, Female, 45 years old)

This sub-theme indicated that PSI forms are available, managers support midwives when they report AEs. However, some view them as punitive, which may explain their reluctance to complete them.

#### Sub-theme 1.2: Supportive incident assessment as a managerial strategy

Incident assessment emerged as a key strategy used by managers to support midwives following AEs. As reported by participants, facilities have established processes for managers to assess incidents by examining the cause, the impact on the patient and any system failures that may have contributed to the event. Proactive managers need to assess why the incident occurred, whether it could lead to litigation, and, in addition, develop measures to prevent future occurrences; this is considered a learning opportunity for quality care. These prescribed assessment processes were confirmed by one of the participants:

‘As managers, we provide support and facilitate a structured investigation following an adverse event to determine the root cause. This includes reviewing the sequence of events, actions taken, associated risks, and the individuals involved or affected. We also assist in classifying the incident as staff- or patient-related and in clinically reviewing records to ensure they are accurately completed, particularly in situations with potential medico-legal implications.’ (Manager 1, Female, 39 years old)

Another participant concurred and noted that reporting must be completed within the prescribed 30-day period, with the quality assurance team involved:

‘A manager must investigate sequences of events and, if necessary, involve the doctor by sharing the information with the patient and family as they gather it. Findings must be compiled, and an incident report must be written about the incident. The whole process must be completed within 30 days of the occurrence, after which it is evaluated by the Quality Assurance team of the Department of Health in the district office to determine whether it will result in litigation.’ (Manager 9, Female, 48 years old)

Another participant confirmed stating:

‘The analysis of the incident will be conducted, and its purpose is to identify any contributing factors or underlying causes. This could include equipment malfunctions or human errors, such as poor record-keeping. Patient records will be reviewed retrospectively to verify that all details were completed. During the analysis of the incident, one must check whether it resulted in harm, whether it was serious or moderate, or whether it ended in a fatal incident and litigation.’ (Manager 4, Female, 55 years old)

The sub-theme highlights the supportive role of managers in assessing AEs by determining their nature, severity and contributing factors, and in identifying measures to prevent recurrence. Through this process, managers also guide midwives in understanding potential medico-legal implications, thereby ensuring they are adequately prepared and supported should litigation arise.

### Theme 2: Psychosocial support strategies

Psychosocial support focuses on enhancing midwives’ emotional and mental well-being. Exposure to AEs can significantly affect individuals, often intensifying post-traumatic stress, particularly in traumatic situations. When providing support, managers must demonstrate active listening when engaging with affected midwives. Managerial psychosocial support helps restore social cohesion and empowers midwives to cope with adversity. Within this theme, two sub-themes emerged: reassurance as a form of emotional support and follow-up, both of which are critical after AEs, as indicated in Table 2.

#### Sub-theme 2.1: Reassurance as a form of emotional support

Managers had to consider the emotional well-being of midwives during AEs. One participant reported that when an AE occurs, midwives show signs of emotional distress and fear that they might lose their jobs. As such, the manager must reassure them, as AEs can sometimes occur because of conditions beyond their control. Support for midwives by managers was confirmed by the following statement:

‘Midwives involved in AEs are very emotional. They always think they will lose their jobs. We call them to a quiet place and reassure them that AEs will always occur where human beings interact, but that we must learn from them. AEs do happen, and it does not mean that you are the cause, as sometimes the patient can be at fault, or they can find themselves in a condition that is out of their control.’ (Manager 9, Female, 48 years old)

Another participant, with a different version, reported that even when they offer emotional support, midwives sometimes do not appreciate their efforts. The same participant continues to state that they are also affected when an AE occurs, as they are required to account for the act. This information was affirmed by the following statement:

‘Being a manager is draining us. We are expected to support our staff members, even when we are not seen as such by our subordinates. We do our best, but we are also frustrated when AEs occur. We are sometimes expected to account to family members when AEs occur, which is very strenuous. The more we try to reassure them and offer emotional support, the less we are appreciated.’ (Manager 12, Female, 44 years old)

Another participant reported that, from experience, if a midwife feels unsupported after an AE, their emotional state escalates to depression, which exposes them to more AEs; they need to be supported after an AE. The following statement is supportive of this fact:

‘As managers, we remain nurses at heart and have a responsibility to care for those who care for others. When midwives feel unsupported, they become emotionally distressed, disheartened, and physically exhausted, increasing their vulnerability to adverse events. Some experience severe emotional strain, including depression. It is therefore essential for managers to provide reassurance, listen actively, and offer consistent emotional support so that midwives feel safe raising concerns without hesitation. Although adverse events may sometimes occur unexpectedly, we strive to reassure midwives that they are not alone and that managerial support is always available to guide and protect them through such challenging moments.’ (Manager 10, Female, 60 years old)

The results of the above-mentioned sub-theme indicate that, although managers are perceived as less supportive, there are positive signs of humanity, suggesting emotional support for midwives from managers.

#### Sub-theme 2.2: Provision of follow-up

Participants reported that once all required post-incident documentation is complete, managers provide follow-up support to assess the affected midwife’s coping mechanisms. This support may include granting time away from the facility or temporarily redeploying the midwife from the unit where the incident occurred. These measures are intended to alleviate stress and provide emotional relief while the potential for litigation against the midwife and the facility is being assessed:

‘After all these findings and report writing, the incident must be verified, and a follow-up must be conducted to see how the midwife is coping and to check whether any harm occurred. As is well known, the mother’s body SANC is responsible for caring for the community; if a midwife is found guilty, they may be struck off the roll, thereby losing their career. It is very difficult to manage such cases, which can emotionally affect midwives. Midwives rely on us for support. We need to conduct a follow-up to review the finer details of the event and demonstrate our care for them. Litigation is not easy; one never knows the outcome. On the other hand, as a manager, you need to monitor the patient’s condition after discharge and protect and support the midwife. As you follow up with the patient, you are simultaneously checking whether they consider taking further steps regarding the event.’ (Manager 5, Female, 47 years old)

Another participant added that, after reconfirming that all aspects of the incident have been addressed, a follow-up is conducted to assess the patient’s impact:

‘Once the PSI forms are completed, there are rules or channels to be followed. When reviewing whether everything is stated and whether there is an indication of future litigation. If not, a follow-up of the incident is conducted to monitor how the affected patient is progressing even after the patient is discharged home.’ (Manager 11, Female, 49 years old)

Managers did their best, although the midwives reported a lack of care and support from them. Midwives’ frustration leads them to overlook the support managers provide following AEs. A participant articulated:

‘A managerial position is not just easy, as you have to check whether all participants are emotionally stable after the occurrence of AEs. We set up a time to check whether all involved parties needed counselling, because you might overlook it and later find that a staff member slips into a depressive state when they are not supported. It is very important to support them.’ (Manager 6, Female, 56 years old)

This sub-theme highlighted the importance of managerial follow-up. Once midwives felt supported, they gained confidence in the management team, as guidance and corrective measures were provided.

### Theme 3: Organisational and capacity-building support strategies

Organisational support and capacity-building are closely interrelated. Supportive strategies include training initiatives that strengthen skills and improve workforce efficiency. Organisational strategies focus on enhancing internal processes and creating a conducive working environment to improve performance. By contrast, capacity-building efforts enhance workers’ competencies and resource utilisation through targeted training to prevent AEs.

#### Sub-theme 3.1: Training of midwives

Participants highlighted that training is a key form of support for midwives, particularly when it includes follow-up to prior sessions, policy implementation evaluations and guidance on Standard Operating Procedures (SOPs) to enhance efficiency and quality of care. They emphasised that monitoring and evaluation should be integrated into training programmes, using lessons from AEs to continually improve midwives’ knowledge and skills in maternal healthcare services. One participant emphasised the importance of continuous professional development for midwives, stating that staying up to date with new developments is essential. The participant explained that training sessions are organised in collaboration with obstetricians to equip midwives to manage emergencies in the unit before a doctor arrives. In-service training is conducted monthly, and legal aspects are incorporated into these sessions to increase midwives’ awareness of factors that may contribute to the recurrence of AEs. The effectiveness of these training sessions is subsequently assessed during ward meetings as part of follow-up activities. The following quote confirms this statement:

‘Research is continuous, and midwives need to be up to date with new developments. We organise training with obstetricians to enable staff to manage emergencies in the unit before a doctor arrives. In-service training is conducted monthly, and legal issues are also addressed during these sessions, helping midwives recognise factors that may contribute to the recurrence of AEs. Assessment of these sessions is conducted during ward meetings as a follow-up to the training.’ (Manager 9, Female, 48 years old)

Another participant added that the in-service department is responsible for briefing nurses on the importance of record-keeping, particularly after an AE. The participants said:

‘In-service training is conducted regularly through the hospital’s in-service department. Emphasis is placed on accurate record-keeping of past events, supported by a monitoring tool. A designated person in charge is required to account for activities within the unit and to submit written reports on incidents, in order to assess whether the actions taken are improving the situation.’ (Manager 11, Female 49, years old)

Orientation for new staff is conducted to familiarise them with the facility’s premises and policies. Quarterly training sessions are organised by the in-service training department to develop skills through development programmes. Managers must ensure that midwives attend all internal courses and sign the attendance register. The participant confirmed by saying:

‘New midwives are orientated for two weeks at our facility to acquaint themselves with the policies and guidelines of the premises. These are distributed to all departments to determine how they are utilised in training. We hold quarterly in-service training for all staff members across multiple sessions to accommodate everyone. Attendance is monitored by signing the register to ensure that everybody has attended.’ (Manager 12, Female 44, years old)

The sub-theme highlights the importance of training as a key managerial support strategy. Although most training activities are facilitated by the in-service department, managers play a critical role by allowing midwives time to attend these sessions. Such training equips midwives, thereby helping prevent and reduce recurrent AEs.

#### Sub-theme 3.2: Creating a conducive environment

Monitoring progress and evaluating care effectiveness should occur in a conducive work environment. Such an environment fosters productivity and supports quality, patient-centred care. In this setting, managers are responsible for creating opportunities that promote professional growth and enhance staff performance. Participants reported collaborating with other stakeholders to ensure patient safety. A participant confirmed this by saying:

‘Following an adverse event, we work collaboratively with the safety and infection control committees to develop and implement safety policies on the wards. Midwives are orientated to these policies to prevent the recurrence of adverse events. This process helps create a safe, supportive, and conducive working environment that enables midwives to work confidently and efficiently after an adverse event.’ (Manager10, Female, 40 years old)

Despite negative accounts of working conditions in public hospitals, some respondents described the environment as good. Managers reported fostering a supportive environment by listening to midwives concerned after an AE, thereby confirming the following statement:

‘We prioritise support through active listening. As a manager, it is essential to meet with the midwife in a quiet, safe environment, recognising that they are often anxious and distressed. Providing time to listen, offering nurturing care, and allowing the midwife to recount the event step by step helps them process the traumatic experience. Although healing may not be immediate, this supportive engagement creates space for emotional relief and the beginning of recovery.’ (Manager 8, Female, 46 years old)

In this sub-theme, a conducive environment, was identified, in which managers collaborated with other departments and made time to listen to midwives as they shared their experiences.

## Discussion

### Clinical support strategies

The findings revealed that managers supported midwives in completing PSI report forms after AEs. Participants reported that managers helped midwives identify which information needed to be documented, including the sequence of events, the severity of the incident and the individuals involved. This support was particularly important, as midwives often experienced fear, anxiety and uncertainty when required to complete PSI forms. These findings align with the literature, which indicates that healthcare professionals’ attitudes towards incident reporting significantly influence reporting behaviour. When reporting is viewed as a learning process rather than a punitive measure, compliance improves (Nkosi 2022; Phillips, Sauzet & Cornelius 2020). However, as in this study, midwives may perceive PSI completion as blame-oriented, leading to reluctance and emotional distress. Liukka et al. (2020) emphasised that a clear understanding of the purpose of incident reporting promotes openness and reduces fear of punishment. The support provided by managers in this study, therefore, played a critical role in reframing incident reporting as a protective and learning mechanism.

The findings further revealed that managers conducted supportive incident assessments by analysing the nature, severity, and causes of AEs while supporting the affected midwife. Managers assessed potential risks, including medico-legal implications, and guided midwives through the assessment process to ensure accurate, fact-based documentation. This supportive approach aligns with evidence that leadership involvement is essential to promoting transparency, learning, and a safety culture following AEs (Phillips et al. 2020). Zoghby, Hoffman and Mahomed (2021) similarly emphasised the importance of structured incident management systems that integrate reporting, assessment, and feedback. By creating a supportive environment during incident assessment, managers in this study reduced anxiety and helped midwives feel protected rather than blamed.

### Psychosocial support strategies

The results indicated that managers provided emotional reassurance to midwives after AEs by actively listening, offering one-to-one support and acknowledging the emotional impact of the incident. Midwives were described as emotionally distressed, fearful and sometimes traumatised after AEs, underscoring the need for compassionate, reassuring managerial support. These findings align with the existing literature, which indicates that psychological safety is critical for encouraging open communication and emotional recovery after clinical incidents (Barry 2024; O’Donovan, Van Dun & McAuliffe 2020). Structured, supportive debriefings enable healthcare professionals to process traumatic experiences and reduce emotional harm (Duff et al. 2024). In maternity care, a lack of reassurance and emotional support following serious incidents has been linked to professional disengagement and psychological injury.

Participants reported that managers provided follow-up support after AEs by monitoring coping strategies, granting time away from the unit, redeploying affected midwives and facilitating referrals for psychological counselling as needed. Managers also followed up with families on behalf of midwives, reducing emotional burden and fear of confrontation. This aligns with evidence that inadequate follow-up contributes to unresolved grief and prolonged emotional distress among healthcare professionals (Raetze et al. 2022). Christoffersen, Teigen and Rønningstad (2020) found that proactive managerial follow-up in maternity settings mitigated trauma experienced by midwives as second victims. Similarly, Ocloo et al. (2021) recommended structured follow-up roles to ensure ongoing engagement and emotional support after AEs. Adams et al. (2023) further demonstrated that consistent follow-up enhances trust, safety outcomes, and reporting culture.

### Organisational and capacity-building support strategies

The study found that managers supported midwives by facilitating training and in-service education on incident management, emergency response, legal issues and SOPs. Follow-up on training content and evaluation of its implementation were also reported as important support mechanisms. Despite these efforts, some midwives continued to view PSI reporting as punitive, underscoring the need for ongoing training and reinforcement. This finding aligns with Liukka et al. (2020), who emphasised that sustained education is necessary to change perceptions and embed a learning-oriented safety culture. Phillips et al. (2020) Mc Carthy et al. 2021 further noted that integrating patient safety tools into routine systems enhances learning and implementation, while Riera et al. (2023) highlighted the role of communication strategies in strengthening organisational policies.

Findings showed that managers fostered a conducive work environment by collaborating with safety and infection control committees, implementing safety policies and orienting midwives to new practices after AEs. Quiet spaces, supportive leadership and clear processes enabled midwives to work more confidently after AEs. Johansen et al. (2021) found that positive practice environments with strong managerial support are associated with improved staff well-being and reporting behaviour. This study confirms that a supportive organisational environment is essential for emotional recovery, professional confidence and preventing recurrent AEs.

### Recommendations

Healthcare organisations should reinforce non-punitive incident reporting through continuous training and clear communication. Managers should be equipped with skills in supportive, trauma-informed leadership. Structured psychosocial follow-up and ongoing capacity-building initiatives should be implemented to promote midwives’ well-being, transparency, and the prevention of recurrent AEs.

### Limitations

The findings of the study were contextual, specifically relevant to district hospitals in Tshwane. The study was confined to one province and three district hospitals, which limits generalisability.

## Conclusion

The study highlights the critical role of managerial support in assisting midwives after AEs. Guidance in PSI reporting and supportive incident assessment reduced fear, anxiety and perceptions of blame by promoting a learning-oriented safety culture. Emotional reassurance, follow-up support and organisational strategies such as training and supportive work environments enhanced midwives’ psychological recovery, professional confidence, and patient safety outcomes.

## References

[CIT0001] Adams, M., Hartley, J., Sanford, N., Heazell, A.E., Iedema, R., Bevan, C. et al., 2023, ‘Strengthening open disclosure after incidents in maternity care: A realist synthesis of international research evidence’, *BMC Health Services Research* 23(1), 285. 10.1186/s12913-023-09033-2 36973796 PMC10041808

[CIT0002] Barry, E., 2024, ‘Leadership and followership within healthcare teams: Exploring the roles and collaborative dynamics in interprofessional teams’. *Doctor of Philosophy*. Maastricht University.

[CIT0003] Bingham, J., Kalu, F.A. & Healy, M., 2023, ‘The impact on midwives and their practice after caring for women who have a traumatic childbirth: A systematic review’, *Birth* 50(4), 711–734. 10.1111/birt.12759 37602792

[CIT0004] Brink, H., Van der Walt, C. & Van Rensburg, G., 2022, *Fundamentals of research methodology for health care professionals*, 5th edn, Juta and Company, Cape Town.

[CIT0005] Cankaya, S., Erkal Aksoy, Y. & Dereli Yılmaz, S., 2021, ‘Midwives’ experiences of witnessing traumatic hospital birth events: A qualitative study’, *Journal of Evaluation in Clinical Practice* 27(4), 847–857. 10.1111/jep.13487 33006235

[CIT0006] Christoffersen, L., Teigen, J. & Rønningstad, C., 2020, ‘Following up midwives after adverse incidents: How front-line management practices help second victims’, *Midwifery* 85, 102669. 10.1016/j.midw.2020.102669 32120330

[CIT0007] Creswell, J.W. & Plano-Clark, V.L., 2023, ‘Revisiting mixed methods research designs twenty years later’, in *Handbook of mixed methods research designs*, vol. 1(1), pp. 21–36, Sage publications.

[CIT0008] Duff, J.P., Morse, K.J., Seelandt, J., Gross, I.T., Lydston, M., Sargeant, J. et al., 2024, ‘Debriefing methods for simulation in healthcare: A systematic review’, *Simulation in Healthcare* 19(1S), S112–S121. 10.1097/SIH.0000000000000765 38240623

[CIT0009] Duffy, J.R., 2022, *Quality caring in nursing and health systems: Implications for clinicians, educators, and leaders*, Springer Publishing Company, Berlin, Germany.

[CIT0010] Evans, T.R., Burns, C., Essex, R., Finnerty, G., Hatton, E., Clements, A.J. et al., 2023, ‘A systematic scoping review on the evidence behind debriefing practices for the well-being/emotional outcomes of healthcare workers’, *Frontiers in Psychiatry* 14, 1078797. 10.3389/fpsyt.2023.1078797 37032950 PMC10080145

[CIT0011] Falcone, M.L., Van Stee, S.K., Tokac, U. & Fish, A.F., 2022, ‘Adverse event reporting priorities: An integrative review’, *Journal of Patient Safety* 18(4), e727–e740. 10.1097/PTS.0000000000000945 35617598

[CIT0012] Flanagan, J. & Beck, C.T., 2024, *Polit & Beck’s nursing research: Generating and assessing evidence for nursing practice*, Lippincott Williams & Wilkins, PA.

[CIT0013] Goekcimen, K., Schwendimann, R., Pfeiffer, Y., Mohr, G., Jaeger, C. & Mueller, S., 2023, ‘Addressing patient safety hazards using critical incident reporting in hospitals: A systematic review’, *Journal of Patient Safety* 19(1), e1–e8. 10.1097/PTS.0000000000001072 35985209 PMC9788933

[CIT0014] Gombau-Giménez, L., Almansa-Martínez, P., Suarez-Cortés, M., Molina-Rodríguez, A., Leal-Costa, C. & Jiménez-Ruiz, I., 2022, ‘Obstetric complications in women from sub-Saharan Africa – A cross-sectional study’, *International Journal of Environmental Research and Public Health* 19(16), 10101. 10.3390/ijerph191610101 36011736 PMC9408375

[CIT0015] Hamdan, A.B., Javison, S., Alharbi, M. & AlHarbi, M., 2023, ‘Healthcare professionals’ culture toward reporting errors in the oncology setting’, *Cureus* 15(4), e38279. 10.7759/cureus.38279 37255901 PMC10226157

[CIT0016] Hamed, M.M.M. & Konstantinidis, S., 2022, ‘Barriers to incident reporting among nurses: A qualitative systematic review’, *Western Journal of Nursing Research* 44(5), 506–552. 10.1177/0193945921999449 33729051

[CIT0017] Houser, J. & Oja, K., 2025, *Nursing research: Reading, using, and creating evidence with navigate advantage access*, Jones & Bartlett Learning, Burlington, MA.

[CIT0018] Isaac, D., Li, Y., Wang, Y., Jiang, D., Liu, C., Fan, C. et al., 2024, ‘Healthcare workers’ perceptions of patient safety culture in selected Ghanaian regional hospitals: A qualitative study’, *BMC Psychology* 12(1), 272. 10.1186/s40359-024-01628-6 38750584 PMC11094925

[CIT0019] Johansen, L.T., Braut, G.S., Acharya, G., Andresen, J.F. & Øian, P., 2021, ‘Adverse events reporting by obstetric units in Norway as part of their quality assurance and patient safety work: An analysis of practice’, *BMC Health Services Research* 21(1), 931. 10.1186/s12913-021-06956-6 34493278 PMC8424984

[CIT0020] Kim, M.J., Jang, S.G., Kim, I.S. & Lee, W., 2021, ‘A study on the status and contributory factors of adverse events due to negligence in nursing care’, *Journal of Patient Safety* 17(8), e904–e910. 10.1097/PTS.0000000000000791 33009180

[CIT0021] Kumah, A., 2025, ‘Adverse event reporting and patient safety: The role of a just culture’, *Frontiers in Health Services* 5, 1581516. 10.3389/frhs.2025.1581516 40910122 PMC12405316

[CIT0022] Liukka, M., Steven, A., Vizcaya Moreno, M.F., Sara-Aho, A.M., Khakurel, J., Pearson, P. et al., 2020, ‘Action after adverse events in healthcare: An integrative literature review’, *International Journal of Environmental Research and Public Health* 17(13), 4717. 10.3390/ijerph17134717 32630041 PMC7369881

[CIT0023] Luo, W., 2025, ‘An empirical study of integration models and mechanisms for maternity care in innovative vocational education’, *Scientific Reports* 16(1), 2053. 10.1038/s41598-025-31951-w 41372377 PMC12808121

[CIT0024] Lou, Y., Zhang, M., Zou, Y., Zhao, L., Chen, Y. & Qiu, Y., 2025, ‘Facilitators and barriers in managing older chronic heart failure patients in community health care centers: A qualitative study of medical personnel’s perspectives using the socio-ecological model’, *Frontiers in Health Services* 5, 1483758. 10.3389/frhs.2025.1483758. 40343233 PMC12060259

[CIT0025] Mulaudzi, F.M., Mulaudzi, M., Anokwuru, R.A. & Davhana-Maselesele, M., 2021, ‘Between a rock and a hard place: Ethics, nurses’ safety, and the right to protest during the COVID-19 pandemic’, *International Nursing Review* 68(3), 270–278. 10.1111/inr.12703. 34551118 PMC8653359

[CIT0026] McCarthy, S.E., Keane, T., Walsh, A., Mellon, L., Williams, D.J., Jenkins, L. et al., 2021, ‘Effect of after-action review on safety culture and second victim experience and its implementation in an Irish hospital: A mixed methods study protocol’, *PLoS One* 16(11), e0259887. 10.1371/journal.pone.0259887 34793495 PMC8601442

[CIT0027] Morobe, R.V., 2024, ‘Development of a model to support midwives experiencing adverse events in obstetric units of the Tshwane District, Gauteng Province’, Doctoral thesis, University of Limpopo.

[CIT0028] Morobe, R.V., Ramavhoya, T.I. & Bopape, M.A., 2024, ‘Midwives’ experiences regarding adverse events in obstetric units of the selected district of Gauteng province, South Africa’, *African Journal of Reproductive Health* 28(10), 17–27. 10.29063/ajrh2024/v28i10.2 39624942

[CIT0029] *National guideline for patient safety incident reporting and learning in the health sector of South Africa – Version 2*, 2022.

[CIT0030] Nchabeleng, M.T., 2022, ‘Challenges experienced by healthcare workers when rendering services at a hospital in Limpopo Province, South Africa’, Doctoral thesis, University of Limpopo.

[CIT0031] Ngene, N.C., Khaliq, O.P. & Moodley, J., 2023, ‘Inequality in health care services in urban and rural settings in South Africa’, *African Journal of Reproductive Health* 27(5), 87–95. 10.29063/ajrh2023/v27i5s.11 37584924

[CIT0032] Nkosi, E.M., 2022, ‘Support program for healthcare professionals involved in adverse events in public hospitals in Gauteng’, Doctoral dissertation, University of the Witwatersrand, Johannesburg.

[CIT0033] Ocloo, J., Garfield, S., Franklin, B.D. & Dawson, S., 2021, ‘Exploring the theory, barriers, and enablers for patient and public involvement across health, social care, and patient safety: A systematic review of reviews’, *Health Research Policy and Systems* 19(1), 8. 10.1186/s12961-020-00644-3 33472647 PMC7816359

[CIT0034] O’Donovan, R., Van Dun, D. & McAuliffe, E., 2020, ‘Measuring psychological safety in healthcare teams: Developing an observational measure to complement survey methods’, *BMC Medical Research Methodology* 20(1), 203. 10.1186/s12874-020-01066-z 32727374 PMC7387873

[CIT0035] Phillips, R., Sauzet, O. & Cornelius, V., 2020, ‘Statistical methods for the analysis of adverse event data in randomised controlled trials: A scoping review and taxonomy’, *BMC Medical Research Methodology* 20, 288. 10.1186/s12874-020-01167-9 33256641 PMC7708917

[CIT0036] Polit, D.F. & Beck, C.T., 2018, *Nursing research: Generating and assessing evidence for nursing practice*, Lippincott Williams & Wilkins, Philadelphia, PA.

[CIT0037] Polit, D.F. & Beck, C.T., 2019, *Nursing research: Generating and assessing evidence for nursing practice*, Lippincott Williams & Wilkins PA.

[CIT0038] Puchert, D., 2025, ‘Patient safety concerns in public hospitals in South Africa’, *Newsday*, viewed 26 November 2025, from https://newsday.co.za.

[CIT0039] Raetze, S., Duchek, S., Maynard, M.T. & Wohlgemuth, M., 2022, ‘Resilience in organization-related research: An integrative conceptual review across disciplines and levels of analysis’, *Journal of Applied Psychology* 107(6), 867. 10.1037/apl0000952 34766796

[CIT0040] Riera, R., De Oliveira Cruz Latorraca, C., Padovez, R.C., Pacheco, R.L., Romão, D.M., Barreto, J.O. et al., 2023, ‘Strategies for communicating scientific evidence on healthcare to managers and the population: A scoping review’, *Health Research Policy and Systems* 21(1), 71. 10.1186/s12961-023-01017-2 37430348 PMC10334604

[CIT0041] Rowland, M. & Adefuye, A., 2022, ‘Human errors and factors that influence patient safety in the pre-hospital emergency care setting: Perspectives of South African emergency care practitioners’, *Health SA Gesondheid* 27, 1798. 10.4102/hsag.v27i0.1798 35548059 PMC9082222

[CIT0042] Saunders, M.M., 2024, ‘A tool to teach and assess clinical nurse specialist student prescribing competency: A pilot study’, *Clinical Nurse Specialist* 38(4), 182–188. 10.1097/NUR.0000000000000825 38889059

[CIT0043] Sidhu, R., Su, B., Shapiro, K.R. & Stoll, K., 2020, ‘Prevalence of and factors associated with burnout in midwifery: A scoping review’, *European Journal of Midwifery* 4, 4. 10.18332/ejm/115983 33537606 PMC7839164

[CIT0044] Thapa, D.R., Ekström-Bergström, A., Krettek, A. & Areskoug-Josefsson, K., 2021, ‘Support and resources to promote and sustain health among nurses and midwives in the workplace: A qualitative study’, *Nordic Journal of Nursing Research* 41(3), 166–174. 10.1177/2057158520988452

[CIT0045] Wain, H., Clarke, D.L. & Wall, S., 2024, ‘A decade-long overview of adverse events in tertiary surgical service in South Africa’, *South African Medical Journal* 114(10), 38–42. 10.7196/SAMJ.2024.v114i10.2035 39508226

[CIT0046] Xu, D., Xu, M., Fang, L., Chen, H., Ye, J. & Huang, C., 2025, ‘Exploring the experience of birth trauma from the midwife’s perspective’, *BMC Pregnancy and Childbirth* 25(1), 1031. 10.1186/s12884-025-08205-8 41053598 PMC12502318

[CIT0047] Zoghby, M.G., Hoffman, D. & Mahomed, Z., 2021, ‘The reporting of adverse events in Johannesburg Academic Emergency Departments’, *African Journal of Emergency Medicine* 11(1), 207–210. 10.1016/j.afjem.2020.10.005 33680743 PMC7910192

